# Malignant fibrous histiocytoma of the urinary bladder as a post-radiation secondary cancer: a case report

**DOI:** 10.1186/1752-1947-5-549

**Published:** 2011-11-10

**Authors:** Thirayost Nimmanon, Poonkiat Ruengpoka

**Affiliations:** 1 Department of Pathology, Phramongkutklao College of Medicine, 315 Ratchawithi Road, Thung Phayathai, Ratchathewi, Bangkok 10400, Thailand; 2 Division of Urology, Department of Surgery, Phramongkutklao Hospital, 315 Ratchawithi Road, Thung Phayathai, Ratchathewi, Bangkok 10400, Thailand

## Abstract

**Introduction:**

Malignant fibrous histiocytomas have been periodically reported as the primary tumor in various organs including the urinary bladder, and is the second most frequent sarcoma of the urinary tract in adults. This report discusses a case of the well established diagnosis of a malignant fibrous histiocytoma of the bladder occurring as a post-radiation cancer after the treatment of a cervical carcinoma. Our findings support those of many previous studies and make the view of the nature of the disease clearer.

**Case presentation:**

We report the case of a 54-year-old Thai woman who had been treated with radiation therapy for cervical cancer, who presented to our facility with urinary incontinence. Initially, our patient was diagnosed as having a high-grade urothelial carcinoma. Subsequent radical surgery rendered the final pathological diagnosis, confirmed histologically and immunohistochemically as malignant fibrous histiocytoma, with clinical and pathological staging of T4b N0 M0. Adjuvant chemotherapy was provided for our patient.

**Conclusions:**

This type of malignancy is very aggressive and easily misdiagnosed due to its rarity. Therefore, in a patient with a prior history of irradiation in the pelvic area, this should be considered as a differential diagnosis to ensure early correct diagnosis and treatment.

## Introduction

While commonly found as a soft tissue tumor and considered as the most common soft tissue sarcoma in adults over the age of 40 years [1], malignant fibrous histiocytomas, or the recently named undifferentiated high-grade pleomorphic sarcomas, are periodically reported as the primary tumor in various organs, including the urinary bladder [2]. Even though it is considered rare in the urinary tract, it is counted as the second most frequent sarcoma of the urinary tract in adults [3]. Information about this tumor is still limited due to its rarity, and several tumors previously reported as malignant fibrous histiocytomas were in fact sarcomatoid urothelial carcinomas. We report a case of malignant fibrous histiocytoma originating from the urinary bladder, presenting as a post-radiation bladder cancer.

## Case presentation

A 54-year old Thai woman presented to our facility with a two-month history of urinary incontinence induced by coughing.

She had a history of cervical squamous cell carcinoma, stage IIb, which was treated 15 years previously with radiation therapy. Her six-month interval follow-up pelvic examination and cervicovaginal PAP smears had revealed negative findings and the disease had been considered as in complete remission by the attending radiologist. She was first referred to our gynecology department to find out the cause of her incontinence. On pelvic examination by a gynecologist, her uterine cervix was found to be atrophic with an extraluminal compression at the anterior vaginal wall, covered by an intact vaginal mucosa. Laboratory investigation results revealed mild hematuria (3 to 5 red blood cells per high-power field) and mild anemia (hemoglobin 10 g/dL, hematocrit 31.1%). Eventually, our patient was referred to our urology department for further management.

**Figure 1:**
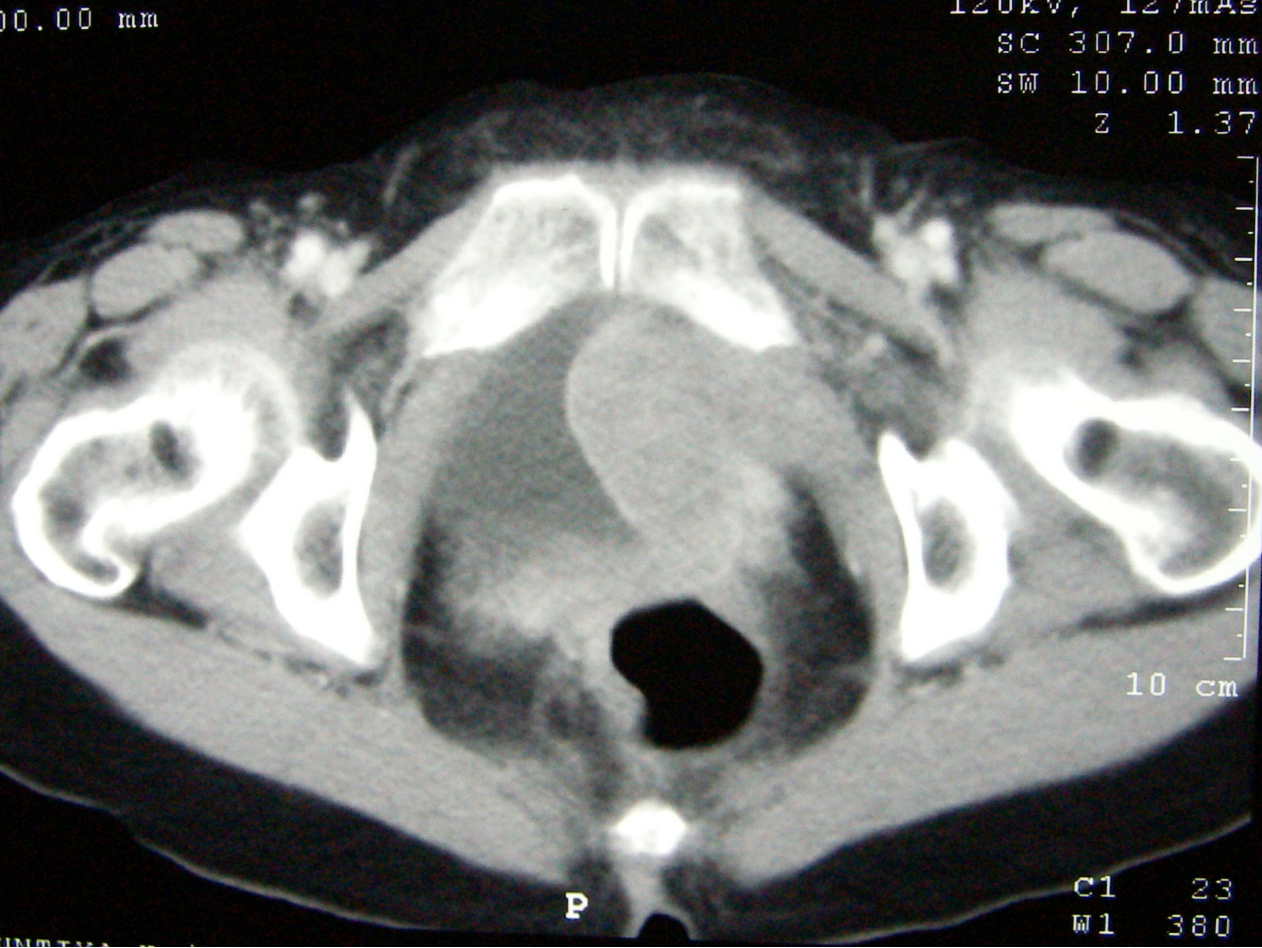
**Computed tomography of the pelvic cavity at the levels of the neoplasm**.

**Figure 2:**
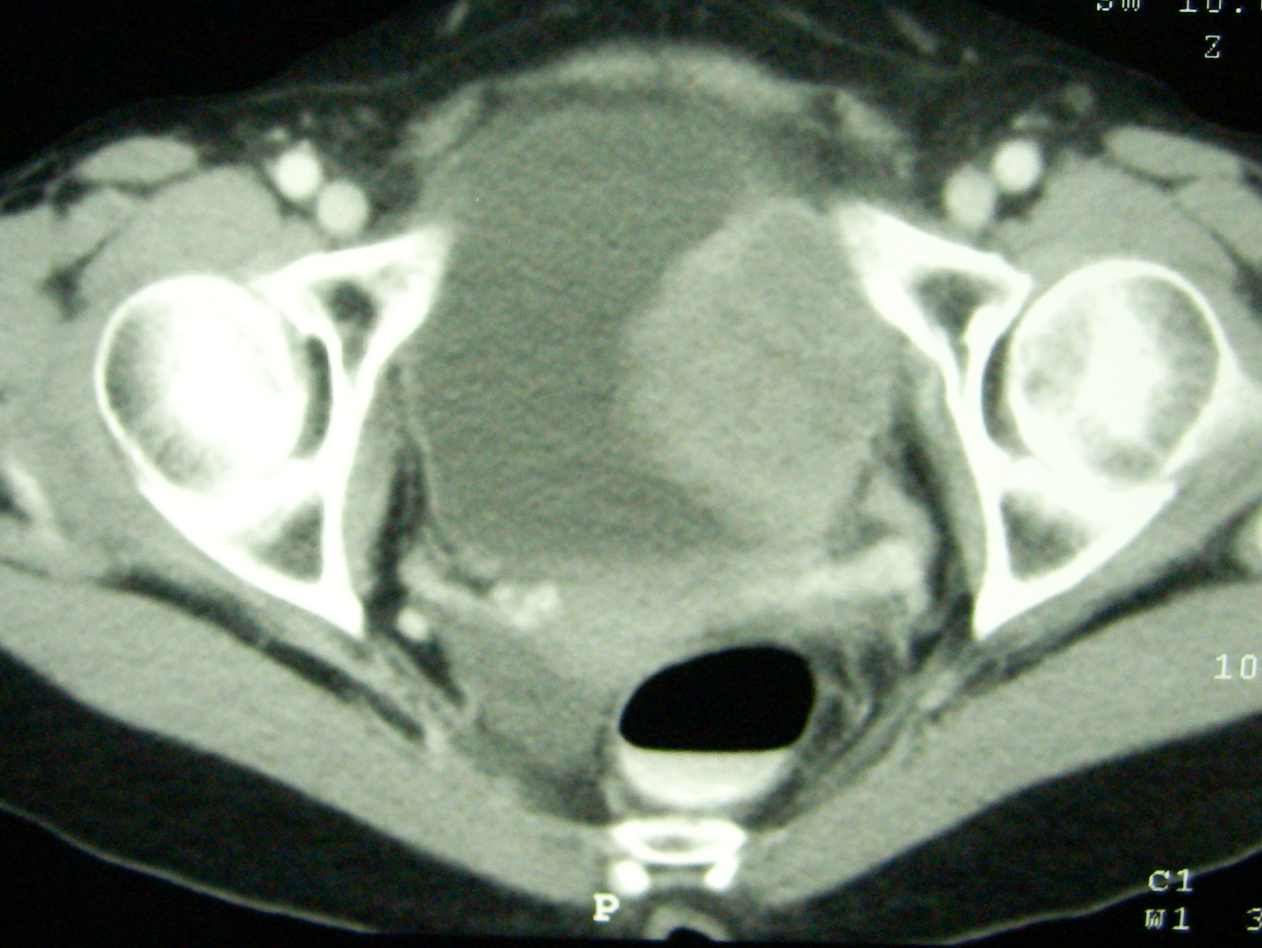
**Computed tomography of the pelvic cavity at the levels of the neoplasm**.

During the process of the Q-tip test, the applicator was passed with difficulty and deviation to the right. Cystoscopy was performed. Her urethra showed no meatal stenosis and unremarkable mucosa, but the device was passed with difficultly due to external compression from the left side of the proximal urethra. Her bladder had a large sessile mass on the left lateral wall with extension to the bladder neck. Both ureteral orifices could be identified clearly with unremarkable appearance and efflux. Clinically, the differential diagnosis included recurrent cervical cancer, bladder cancer and retroperitonal sarcoma. Urine cytology and transurethral resection of the bladder tumor were performed, with a negative cytology report and a pathological diagnosis of high-grade urothelial carcinoma. A computed tomography scan demonstrated a heterogenous enhancing mass, 5.3 × 6.5 × 6.0 cm, at the left inferior-lateral wall of the bladder with invasion of the uterus and attachment to the left pelvic side wall, as shown in Figures 1 and 2. There was no hydronephrosis, hydroureter, liver mass or lymphadenopathy seen radiologically. Subsequently, anterior pelvic exenteration and ileal conduit with pelvic lymphadenectomy, as well as an incidental appendectomy, were performed.

Gross pathological examination revealed a large mass on the left lateral wall that measured 7.0 cm across its greatest dimension. The surface of the tumor on the mucosal side was smooth. The mass obviously protruded from the serosal surface of the bladder and was attached to the uterus, but could still be separated from the organ with ease.

**Figure 3:**
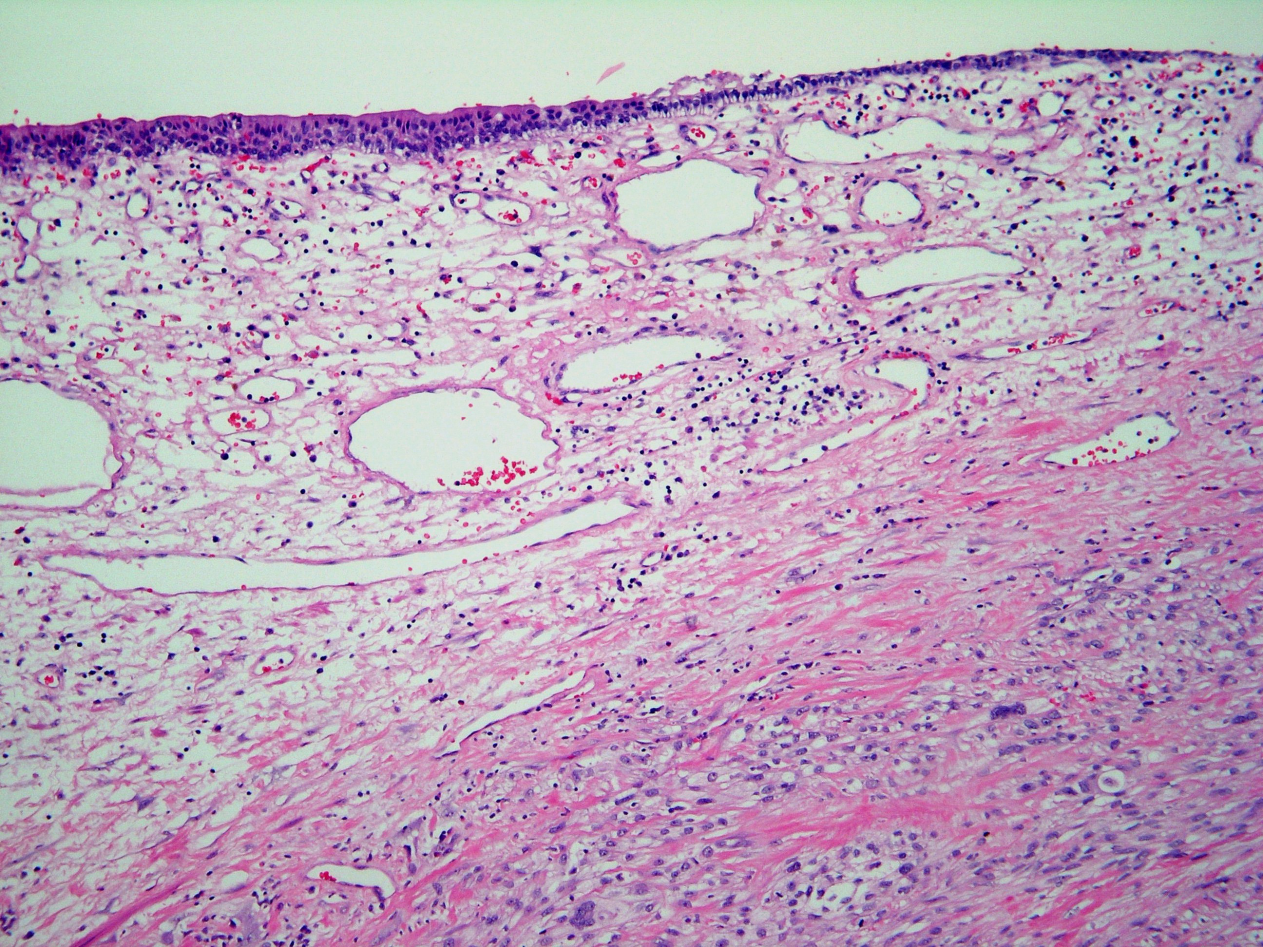
**The intact urothelial lining with underlying malignant neoplasm (low magnification)**.

**Figure 4:**
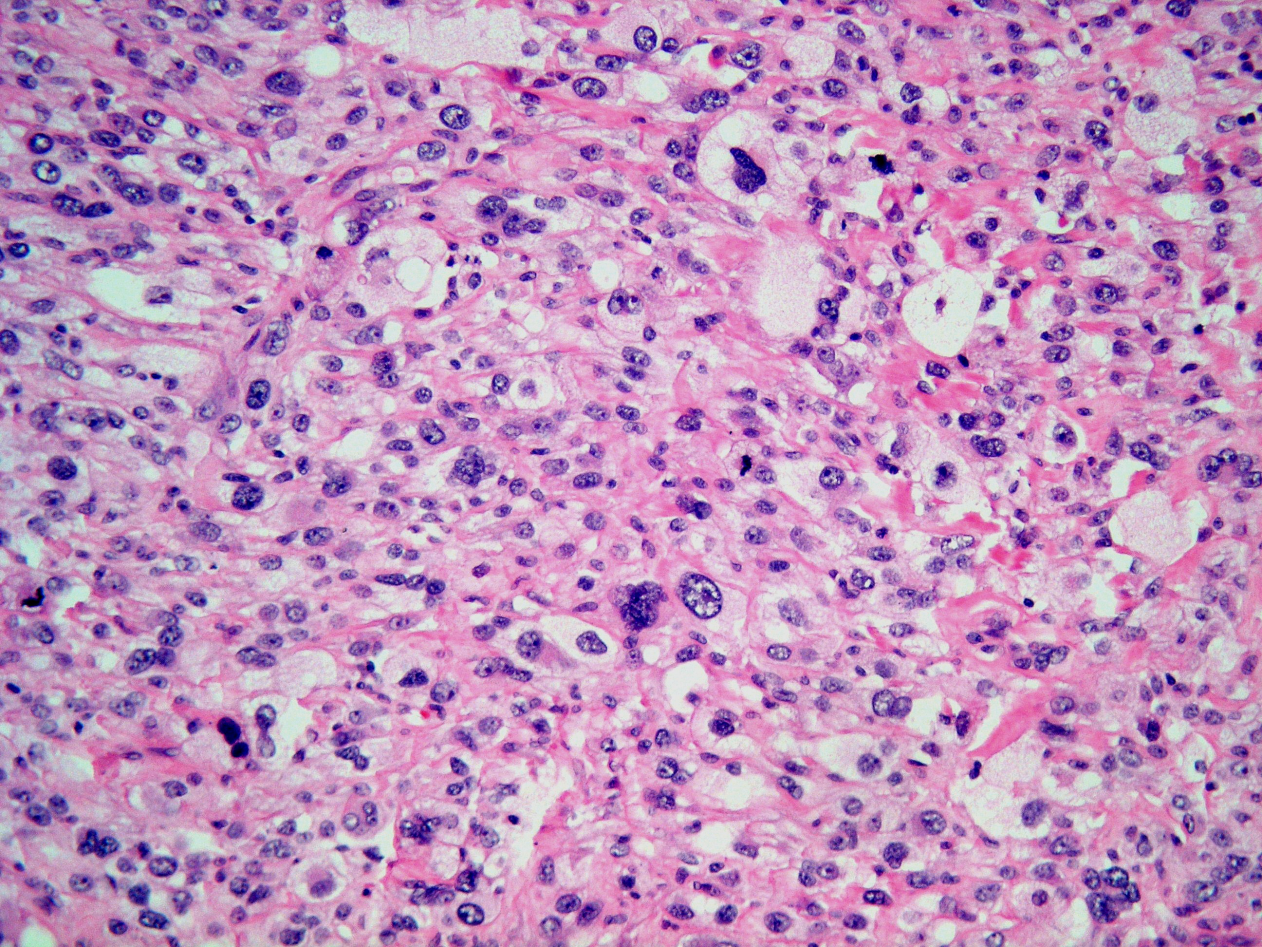
**Highly pleomorphic neoplastic cells with clear to pale eosinophilic cytoplasm (higher magnification)**.

**Figure 5:**
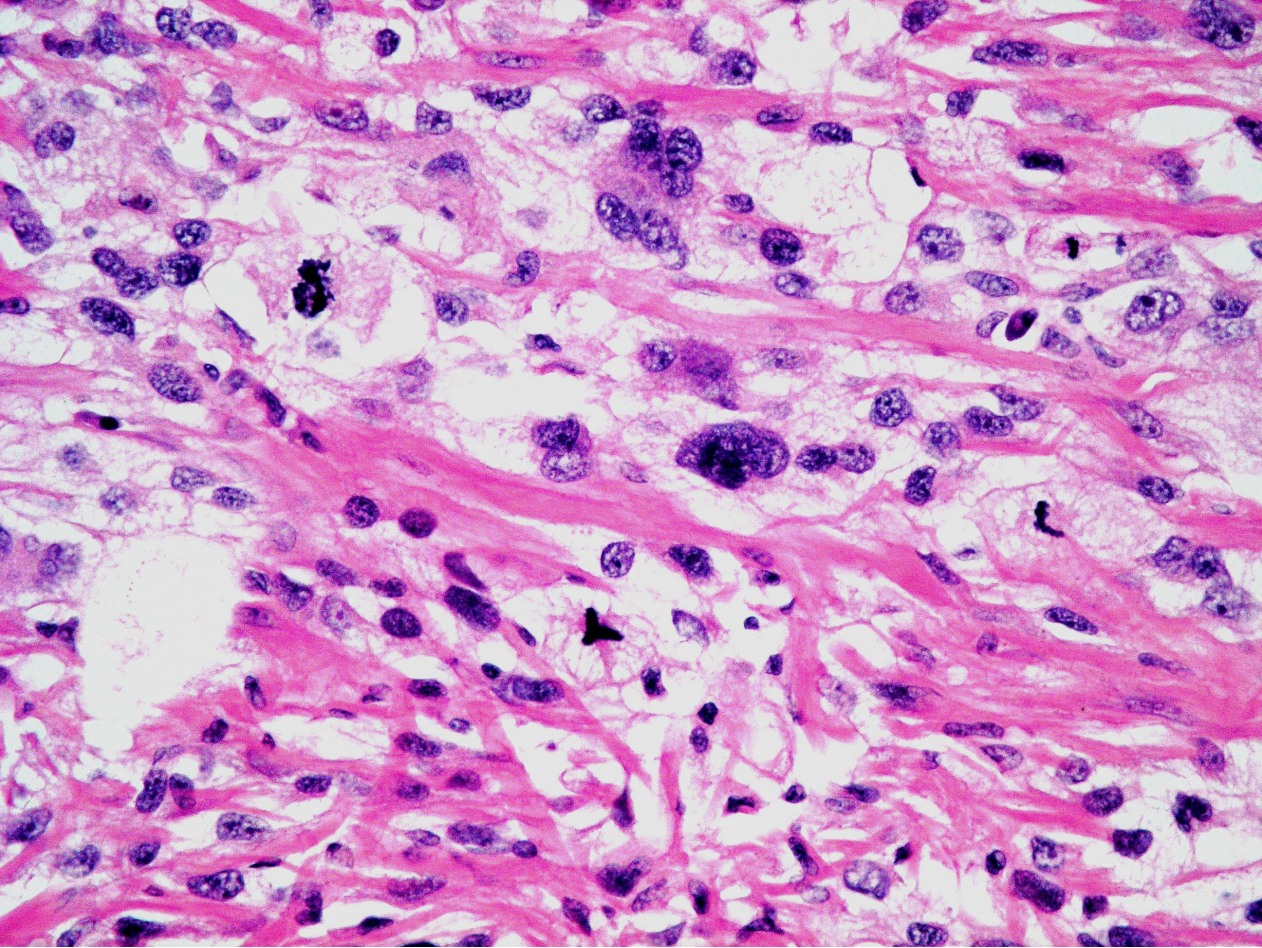
**Some cells contained bizarre-shaped nuclei and some showed atypical mitotic figures**.

**Figure 6:**
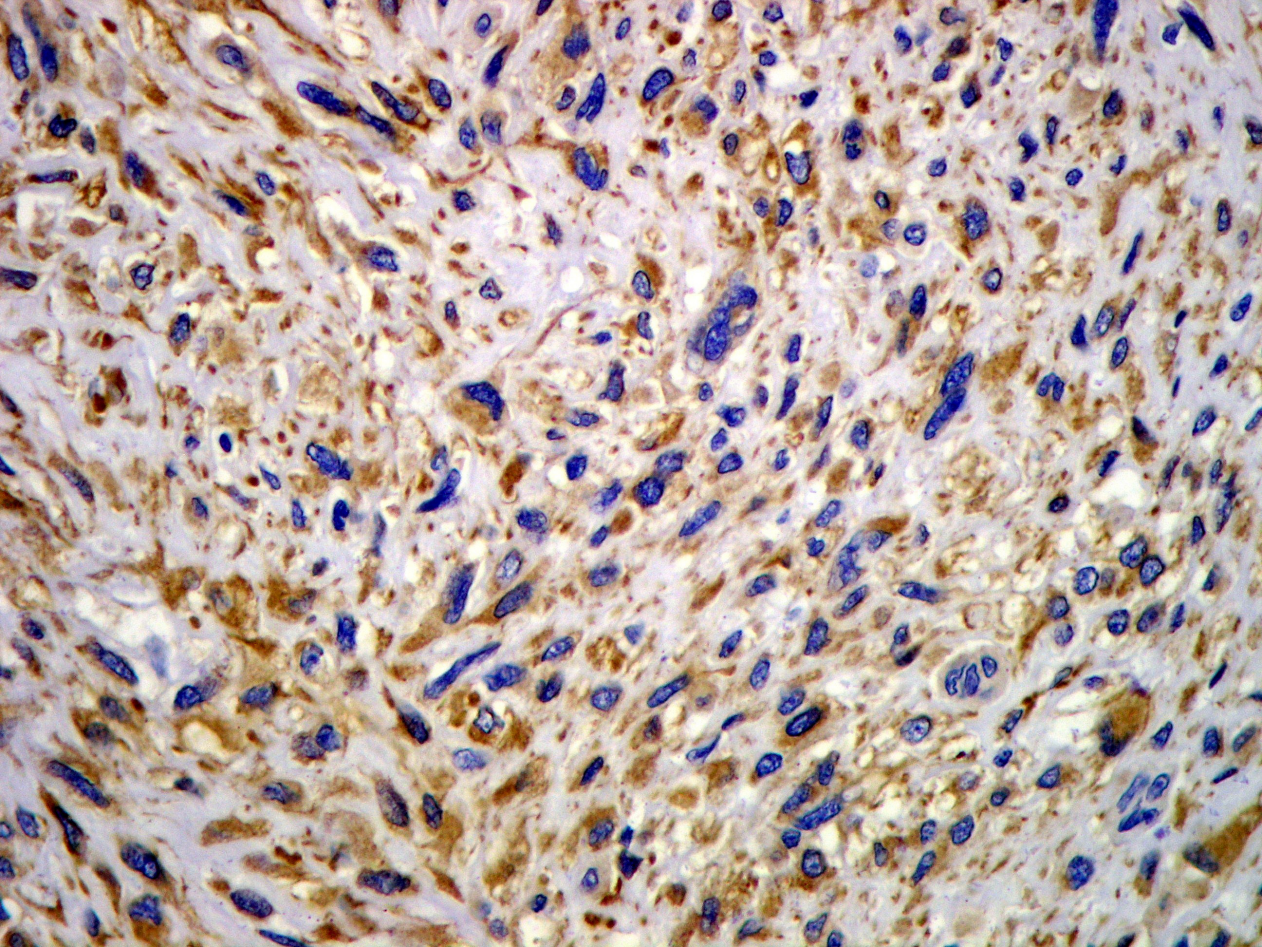
**Immunohistochemical study results showing that the neoplasm was immunoreactive for vimentin**.

**Figure 7:**
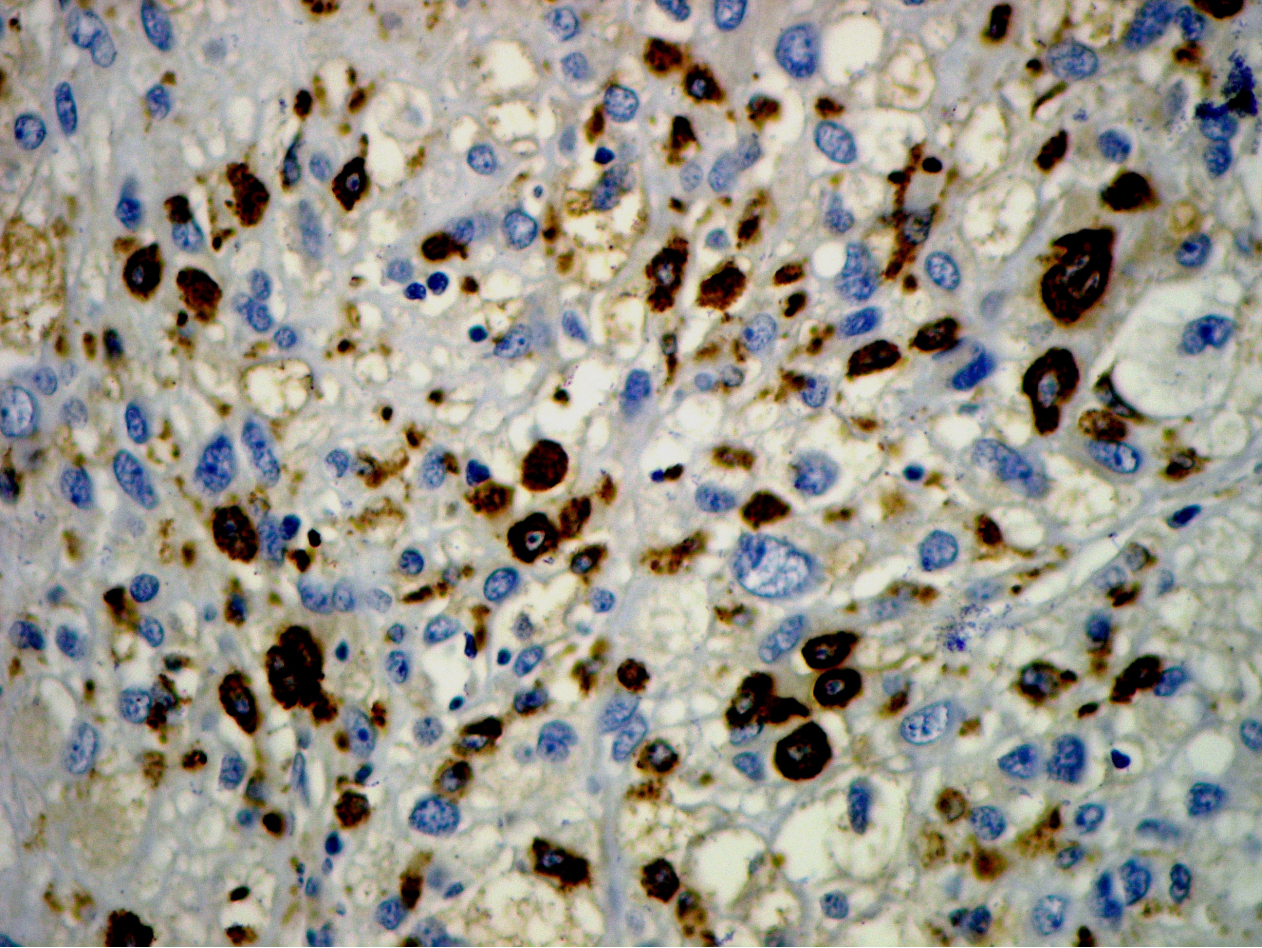
**Immunohistochemical study results showing that the neoplasm was immunoreactive for CD68**.

Microscopically, the overlying urothelium was intact with no dysplastic change (Figure 3). The underlying mass was composed of highly pleomorphic spindle-to-polygonal shaped neoplastic cells with frequent mitotic figures and many atypical ones (Figures 4 and 5). Their cytoplasms ranged from clear to pale on eosinophilic staining. Many cells contained bizarre-shaped nuclei with focal multinucleated giant cells. No definite component of urothelial carcinoma was seen. An immunohistochemical study was performed, showing that the neoplasm was immunoreactive for vimentin (Figure 6) and CD68 (Figure 7), but negative for epithelial marker (AE1/AE3) and other markers for soft tissue tumors (desmin, smooth muscle actin and S-100 protein). A CD10 test was also performed for exclusion of carcinoma of renal cell origin, and yielded a negative result. Therefore, a diagnosis of malignant fibrous histiocytoma was made.

Pathological findings from other organs, including the uterine cervix, were unremarkable. No residual cervical dysplasia or neoplasm was detected. All 12 lymph nodes showed no neoplasm. The disease was clinically and pathologically staged as T4b N0 M0.

After surgery, our radiation therapist advised not providing additional radiation therapy in the pelvic area. Our patient, therefore, received a course of adjuvant chemotherapy (doxorubicin and ifosfamide). However, two months after the surgery, a bony metastasis was detected at the L5 spine level, and palliative radiation therapy in that area was suggested. Despite aggressive surgical treatment along with adjuvant chemotherapy and palliative radiation therapy, our patient developed further locally recurrent disease with invasion to the right pubic bone and rectum. At this time, a multidisciplinary approach for palliation of our patient's symptoms is still in process.

## Discussion

Our patient had received a full course of radiation therapy in her pelvic area and, hypothetically, the bladder sarcoma developed from radiation-induced genetic alterations. The mass developed rapidly and behaved aggressively.

From her clinical history, for purposes of retrospective study, our patient's presentation was not typical for a conventional urothelial carcinoma, as the mass was large while producing negative urine analysis and negative urine cytology results. Even though squamous cell carcinoma of the bladder is strongly associated with chronic irritation or inflammation, it is still unlikely due to the laboratory results and no strong association with radiation has been reported. The history of radiation exposure produced a suspicion of mesenchymal tumor origin. The reported incidence of post-irradiation sarcoma ranges from some few per thousand to nearly 1% [4]. However, most cases have been seen in patients with breast cancer who received adjuvant radiation therapy, and the sites of sarcomas are usually soft tissue in origin. Even though her history indicated that the biopsy diagnosis was high-grade urothelial carcinoma, the diagnosis was reasonable, in our opinion, because of the much higher incidence of urothelial carcinomas, frequently found sarcomatoid components in high-grade urothelial carcinomas, and limited clinical information available. From gross pathological examination, the mass was obviously unlikely for carcinomas, as tumors at this large size usually present as a bulky mass, often with necrosis, and should not have a smooth surface. Therefore, suspicion of sarcomas or lymphomas should be raised.

Few cases of malignant fibrous histiocytoma of the urinary bladder have been reported so far [2]. After a complete international medical literature search, one literature review showed that there had been 29 cases reported up to 2010, and the tumors appear mostly in men (4:1) with a mean age of 60 years (20 to 84), and usually manifest as macroscopic hematuria or irritative urinary symptoms [5]. As in our patient's case, some authors have found an association between malignant fibrous histiocytoma and radiation therapy [6].

Many authors have reported that this type of malignancy is very aggressive, with a high local recurrence rate and a requirement for aggressive treatment with a combination of radical surgery and systemic chemotherapy [5,7,8], while some authors have questioned whether radical oncosurgery is justified for the treatment of primary malignant fibrous histiocytoma of the urinary bladder or not due to its dismal outcome in spite of the early stage of diagnosis [9].

## Conclusions

In summary, malignant fibrous histiocytoma is considered as a rare malignancy of the urinary bladder. However, some of the previously diagnosed high-grade carcinomas might actually be this type of sarcoma. We suspect that the radiation exposure might be a contributing factor to this rare mesenchymal neoplasm. Correct diagnosis requires immunohistochemical studies with a large panel of antibodies in close correlation with histological and cytological features [10].

## Consent

Written informed consent was obtained from the patient for publication of this case report and any accompanying images. A copy of the written consent is available for review by the Editor-in-Chief of this journal.

## Competing interests

The authors declare that they have no competing interests.

## Authors' contributions

PR analyzed and interpreted the data from our patient regarding the neoplasm, and performed the operation. TN performed the histological examination and interpretation of immunohistochemical study results, and was a major contributor to writing the manuscript. All authors read and approved the final manuscript.
